# 

**Published:** 1850-11

**Authors:** 


### WESTERN JOURNAL

OF

### MEDICINE AND SURGERY.

### EDITORS.

---

**LUNSFORD P. YANDELL, M. D.**
PROFESSOR OF PHYSIOLOGY AND PATHOLOGICAL ANATOMY IN THE UNIVERSITY OF LOUISVILLE.

AND

**THEODORE S. BELL, M.D.**

### *Receipts of Medical Journal for October*, 1850.

| | |
|---|---|
| Dr. J. Pollock, Germantown, Ky., | $5 00 |
| M. M. Maughas, Danville, Mo., | 10 00 |
| J. C. Clark, Anderson's Store, Ohio, | 10 00 |
| A. M. Ayres, Salem, Miss., | 10 00 |
| —— Robertson, Jefferson county, Ky., | 5 00 |
| W. B. Williams, Empire Iron Works, Ky. | 5 00 |
| M. H. Fee, Heltonsville, Ind., | 5 00 |
| S. Coon., Dycusburg, Ky. | 5 00 |
| F. H. Gordon, Lebanon, Tenn., | 10 00 |

